# Ulcère de cornée chronique révélant un syndrome de Parry-Romberg: à propos d´un cas

**DOI:** 10.11604/pamj.2021.38.53.27190

**Published:** 2021-01-18

**Authors:** Sara El Maroufi, Ahmed Bennis, Fouad Chraibi, Meriem Abdellaoui, Idriss Benatiya Andaloussi

**Affiliations:** 1 Service d´Ophtalmologie, Centre Hospitalier Universitaire Hassan II, Université Sidi Mohamed Ben Abdellah, Faculté de Médecine et de Pharmacie, Fès, Maroc

**Keywords:** Ulcère de cornée chronique, syndrome de Parry-Romberg, atrophie hémifacial, à propos d’un cas, Chronic corneal ulcer, Parry-Romberg´s syndrome, hemifacial atrophy, case report

## Abstract

Le syndrome de Parry-Romberg, est une entité clinique rare, il est caractérisé par une atrophie hémifaciale progressive et associé à diverses manifestations systémiques, notamment ophtalmologiques, neurologiques et maxillo-faciales dont la prise en charge doit être multidisciplinaire. A travers cette observation nous rapportons le cas d´un syndrome de Parry-Romberg diagnostiqué chez un patient adressé pour prise en charge d´un ulcère de cornée chronique suite à une hypoesthésie selon une atteinte rare et difficile à traiter.

## Abstract (en)

Parry-Romberg´s syndrome is a rare clinical entity characterized by progressive hemifacial atrophy associated with several systemic manifestations including ophthalmologic, neurologic, maxillofacial symptoms whose treatment should be multidisciplinary. We here report a case of Parry-Romberg´s syndrome diagnosed in a patient referred for management of chronic corneal ulcer following hypoesthesia, characterized by rare and difficult-to-treat features.

## Introduction

Le syndrome de Parry-Romberg (PRS), également connu sous le nom d'atrophie hémifaciale progressive, a été décrit pour la première fois par Parry en 1825. Il s'agit d'une maladie rare caractérisée par une atrophie faciale unilatérale affectant la peau, les tissus sous-cutanés, les muscles et les os. Elle a été associée à diverses manifestations systémiques, notamment ophtalmologiques, neurologiques et maxillo-faciales. Les troubles ophtalmiques comprennent la kératite, l'uvéite, la cataracte, l'énophtalmie homolatérale, la névrite optique et la vascularite rétinienne [1].

## Patient et observation

**L´histoire clinique:** il s´agit d´un patient âgé de 31 ans ayant comme antécédent une migraine évoluant depuis 15 ans sans autre symptôme neurologique, qui a été référé à notre service pour prise en charge d´une kératite chronique de l'œil gauche sans contexte traumatique évoluant depuis 3 mois.

**Figure 1:**
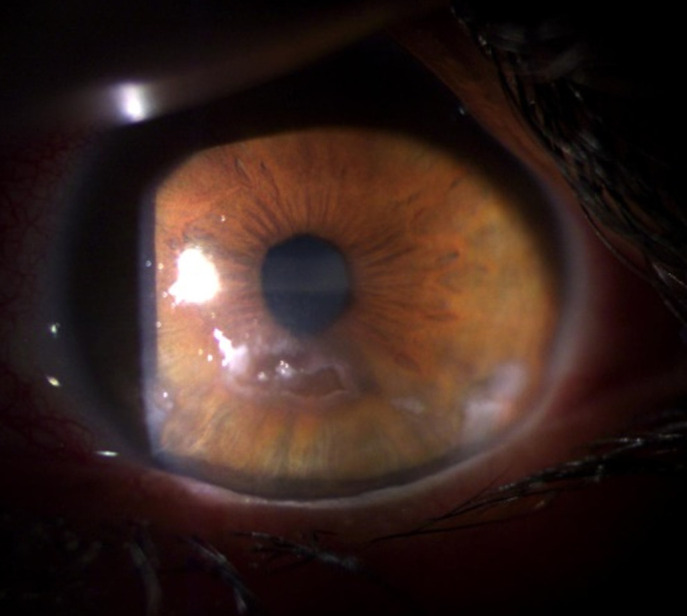
hypoplasie de l´hémiface gauche avec énophtalmie

**L´examen clinique:** l´examen ophtalmologique a objectivé une meilleure acuité visuelle corrigée (BCVA) était de 0,4. L'examen à la lampe à fente a révélé une hyperémie conjonctivale modérée avec un ulcère cornéen infiltré para-central inférieur de 2 mm de large sur 3 mm de haut, avec abcèdation des berges (Figure 1). La sensibilité cornéenne était diminuée et le test de Schirmer altéré à 5mm. Aucune réaction inflammatoire de la chambre antérieure n'a été observée. L´examen de l´œil droit était normal.

**Figure 2:**
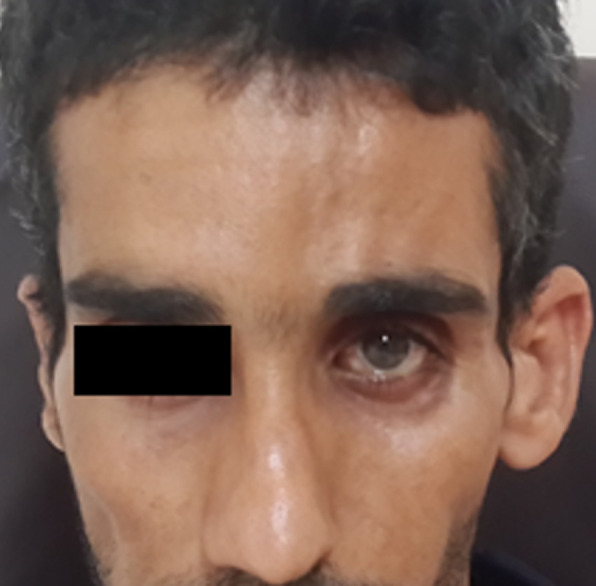
ulcère de cornée avec abcédation des berges

L'examen général a révélé une atrophie hémifaciale (Figure 2), aucun trouble systémique n'a été observé.

**Le traitement:** le patient a été mis sous larmes artificielles, vitamine A pommade avec une antibiothérapie topique (quinolone). Un traitement analgésique pour les crises de migraine a également été prescrit.

**Figure 3:**
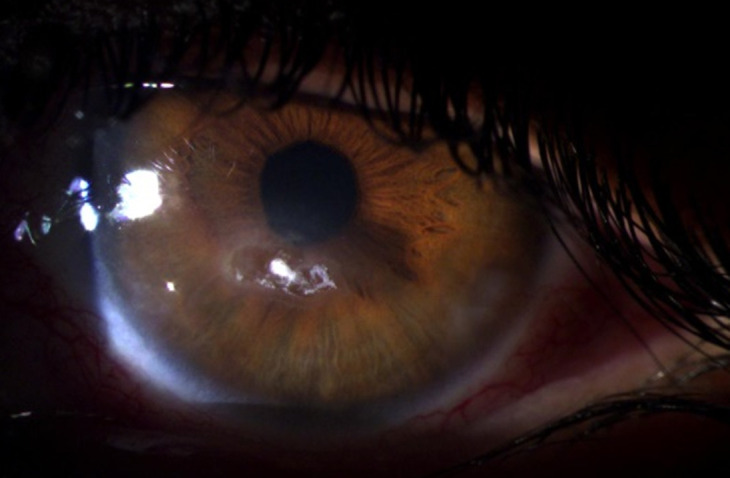
régression de l´abcès après deux jours de traitement

**Figure 4:**
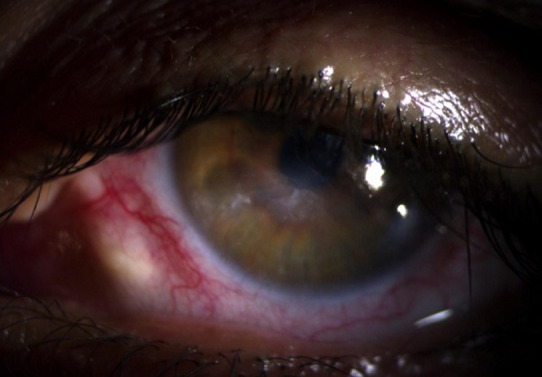
cicatrisation complète après six mois d´évolution

**Suivi clinique et évolution du patient:** l'évolution a été lente avec régression de l´abcès après deux jours de traitement (Figure 3), un nettoyage de l´abcès et début de cicatrisation après trois semaines. La guérison complète a été obtenue après 6 mois de traitement (Figure 4).

## Discussion

Le syndrome de Parry-Romberg est une maladie rare qui se manifeste par une hémiatrophie faciale et une perte de graisse sous-cutanée du côté affecté. On estime qu'une naissance sur 700 000 présente ce syndrome. À ce jour, environ 150 cas de syndrome de Parry-Romberg avaient été rapportés dans la littérature. Il se présente généralement initialement chez les enfants et les jeunes adultes et progresse lentement sur un cours très variable allant de 2 à 20 ans, et se stabilise brusquement sans raison apparente [2, 3]. Il existe plusieurs théories sur l'étiologie du syndrome de Parry-Romberg, notamment la névrite du trijumeau, cette théorie est également appuyée par l´étude de Bucher *et al*. en 2015 utilisant in vivo la microscopie confocale cornéenne permettant parfois d´objectiver une atrophie du nerf trijumeau (V) du coté atteint comparé au côté sain [4].

De nombreuses autres hypothèses tentent d´expliquer l´origine de ce syndrome mais aucune d´entre elle n´est cependant formelle et l´incertitude règne toujours comme la neuro-vascularite auto-immune chronique, l´étiologie infectieuse a aussi été évoquée dans la littérature, les facteurs génétiques, l'auto-immunité et l´activité nerveuse sympathique accrue déclenchant une atrophie faciale [5].

Le syndrome de Parry-Romberg est une maladie auto-limitable sans remède. Les patients atteints devraient avoir une présence multidisciplinaire de chirurgien plasticien, de chirurgien-dentiste, de phono-audiologistes. De nos jours, les chirurgies esthétiques avec greffe de graisse autogène, les injections de silicone ou de collagène bovin et les implants inorganiques sont des alternatives pour corriger les déformations. Outre l'amélioration esthétique, un traitement symptomatique de chaque association systémique selon ses manifestations est nécessaire [3].

Le syndrome de Parry-Romberg coexiste souvent avec d'autres troubles; l'atteinte neurologique est l'association la plus courante, y compris la migraine, l'hémiplégie, l'atrophie cérébrale et l'anomalie vasculaire intracrânienne. Les crises partielles sont la complication neurologique la plus courante [6]. Dans notre cas, la migraine était le seul symptôme neurologique présenté par le patient. Plusieurs associations ophtalmologiques ont été décrites, touchant 10 à 30% des patients, les plus fréquentes sont: l´énophtalmie, l´atrophie des paupières, l´uvéite, la vascularite rétinienne (Tableau 1) [3, 7]. Notre patient avait une énophtalmie et des kératites récurrentes dues à un syndrome sec et à une hypoesthésie, cette atteinte est rare et difficile à traiter.

**Tableau 1:** manifestations oculaires du syndrome de Parry-Romberg [6]

Structure oculaire	Manifestations ophtalmologiques
Orbite	Enophtalmie (notre cas)
Muscles oculomoteurs	Paralysie du III, esotropie, exotropie, diplopie
Paupières	Pseudo-ptôsis, lagophtalmie, rétraction
Cornée	Kératopathie en bandelette, kératite d´exposition, hypoesthésie (notre cas), précipités endothéliales
Iris /uvée	Atrophie de l´iris, hétérochromie de Fuchs, uvéite
Cristallin	Cataracte
Corps ciliaire	Glaucome, hypotonie
Rétine	Vascularite, occlusion de la veine centrale de la rétine
Papille optique	Papillite, neurorétinite, syndrome de Horner

## Conclusion

Le syndrome de Parry Romberg est une maladie rare. Le cas rapporté illustre une manifestation oculaire rare décrite dans la littérature correspondant à la kératite rebelle et récurrente, qui a évolué favorablement sous lubrifiants topiques.
